# The relationship between clinical features and treatment options in sigmoid volvulus: Experience of 59 years and 1,096 patients

**DOI:** 10.12669/pjms.41.10.12899

**Published:** 2025-10

**Authors:** Enes Agirman, Rifat Peksoz, Esra Disci, Sabri Selcuk Atamanalp

**Affiliations:** 1 Enes Agirman, MD Assistant Professor, Department of General Surgery, Erzurum City Hospital, Erzurum, Turkiye; 2 Rifat Peksoz, MD Associate Professor, Department of General Surgery, Faculty of Medicine, Ataturk University, Erzurum, Turkiye; 3 Esra Disci, MD Associate Professor, Department of General Surgery, Faculty of Medicine, Ataturk University, Erzurum, Turkiye; 4 Sabri Selcuk Atamanalp, MD Professor, Department of General Surgery, Faculty of Medicine, Ataturk University, Erzurum, Turkiye

**Keywords:** Sigmoid volvulus, Clinical features, Nonoperative detorsion, Emergency surgery

## Abstract

**Objectives:**

In sigmoid volvulus (SV), nonoperative detorsion is the first treatment option, while some complicated patients require emergency surgery. The purpose of this study was to utilize the clinical features requiring emergency surgery in SV.

**Methodology:**

Among total 1,096 patients with SV, the records of 813 cases (74.2%) treated with nonoperative detorsion and 492 patients (44.9%) required emergency surgery were evaluated in a partial retrospective and prospective scanning.

**Results:**

Previous volvulus history was more common (34.7% vs. 26.2%, respectively, p<0.005), mean symptom period was shorter (23.9 hours vs. 47.5 hours, respectively, p<0.001), the rates of vomiting (62.7% vs. 75.2%, respectively, p<0.001), hypokinetic/akinetic bowel sound (37.3% vs. 48.0%, respectively, p<0.001) and shock (1.3% vs. 22.2%, respectively, p<0.001) were lower, while the rates of hyperkinetic bowel sound (35.3% vs. 20.1%, respectively, p<0.001) and empty rectum (67.9% vs. 60.2%, respectively, p<0.05) were higher in nonoperative detorsion group when compared with that of emergency surgery group. The rates of pregnancy (5.4% vs. 5.6%, respectively, p>0.05), comorbidity (31.7% vs 29.1%, respectively, p>0.05), abdominal pain/tenderness (98.9% vs. 99.0%, respectively, p>0.05), distention (97.2% vs. 98.4%, respectively, p>0.05) and obstipation/constipation (92.9% vs. 94.7%, respectively, p>0.05) were similar in both groups. All patients with gangrenous stool (19.5%) and rebound tenderness/muscular rigidity (16.5%) required emergency surgery.

**Conclusion:**

Some preoperational parameters including first attack, prolonged preoperative period, vomiting, hypokinetic/akinetic bowel sound and shock may be relative indicators, while gangrenous stool and rebound tenderness/muscular rigidity are absolute indications of emergency surgery in SV.

## INTRODUCTION

Sigmoid volvulus (SV) is the third most common cause of all large bowel obstructions around the world.^1^ Its incidence is 1.67 cases per 100,000 persons per year in United States, while it is more common in Eastern Anatolia with an incidence of 4.2 per 100,000 person-years.^2^ Abdominal pain/tenderness, distention, obstipation/constipation, vomiting, abnormal bowel sounds, peritoneal irritation findings and hypotension/shock are common symptoms and signs of SV.^1^ Preliminary treatment is nonoperative detorsion (preferably flexible endoscopy) in uncomplicated patients, while cases with bowel gangrene, peritoneal irritation findings, or those with unsuccessful nonoperative attempt require emergency surgery (detorsion, fixation, or preferably colectomy).^3^

In this article, to determine the effects of clinical features on treatment options in SV, we evaluated the clinical records of total 1,096 patients. According to a data search of the last 80 years of literature (from 1945 to 2025) in Web of Science database,^4^ our series constitutes the largest published single-center SV data over the world.

## METHODOLOGY

Among total 1,096 patients with SV, we used partial retrospective (612 patients, 55.8%, from June 1966 to July 1986) and prospective (484 cases, 44.2%, from July 1986 to July 2025) evaluations. We used nonoperative detorsion (barium enema, rigid or flexible endoscopy) as first line option in uncomplicated cases, while we required emergency surgery (detorsion, mesopexy, exteriorization or sigmoidectomy) in patients with gangrenous stool, peritoneal irritation findings (rebound tenderness/muscular rigidity) or diagnostic problems, or in childhood. Additionally, we used emergency surgery in patients with gangrenous bowel in nonoperative attempt, unsuccessful nonoperative detorsion or early SV recurrence following successful nonoperative detorsion.

Previous volvulus history, pregnancy, comorbidity, symptom period, symptoms and signs including abdominal pain/tenderness, distention, obstipation/constipation, vomiting, bowel sounds, peritoneal irritation findings (rebound tenderness/muscular rigidity), digital rectal examination findings (empty rectum or gangrenous stool) or shock were noted in each case.

In the evaluation, patients with early SV recurrence were excluded from those with successful nonoperative detorsion and the remained cases were accepted as nonoperative detorsion group. On the other hand, patients treated with emergency surgery as preliminary treatment or second to nonoperative detorsion (unsuccessful nonoperative detorsion, gangrenous bowel in nonoperative detorsion, or early SV recurrence following successful nonoperative detorsion) were accepted as emergency surgery group.

In statistical analyses, we preferred IBM SPSS 20 program. We presented the data as mean, standard deviation, number and percentage. We used Shapiro Wilk test and Kolmogorov Smirnov test in the analysis of normal distribution of continuous variables. We preferred Independent Samples t test or Mann Whitney u test in the comparison of independent two groups, while Chi-square test was used in the evaluation of two percentages. We set statistical significance level as p<0.05. Institutional review board (Ethical Committee of Ataturk University Faculty of Medicine, 2025/03) and written informed consent forms were obtained.

## RESULTS

**Fig.1:**
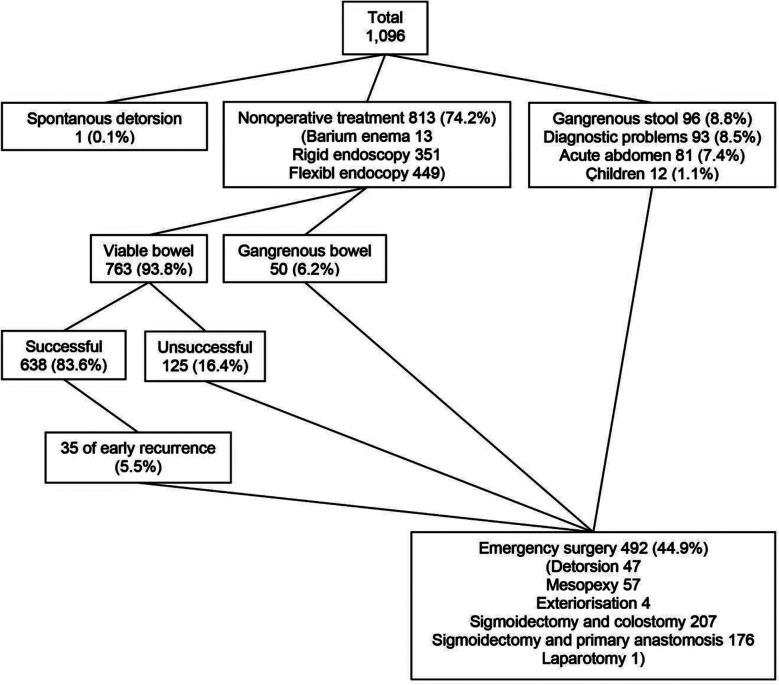
Treatment options in patients with sigmoid volvulus.

Treatment options in patients with SV are summarized in Fig.1, while treatment options in relation to clinical features and statistical analyses are given in Table-I. Among total 1,096 patients with SV, nonoperative detorsion was tried in 813 cases (74.2%, 13 barium enema, 351 rigid endoscopy and 449 flexible endoscopy) with 83.6% of success rate (638 patients among 763 cases with viable bowel). When 35 cases (5.5%) treated with emergency surgery among those with early SV recurrence were excluded, total 603 cases benefited from nonoperative detorsion. In this cohort, we evaluated the records of 533 cases (88.4%), in whom the records were found. Emergency surgery was applied as preliminary treatment in 282 patients (25.7%, 96 gangrenous stool, 93 diagnostic problems, 81 acute abdomen and 12 children), while it was used following nonoperative detorsion in 210 cases (19.2%, 125 unsuccessful nonoperative detorsion, 50 gangrenous bowel in nonoperative detorsion and 35 early SV recurrence). In this way, emergency surgery was required in total 492 patients (44.9%) and we evaluated all of surgically treated cases.

**Table-I:** Clinical features and treatment options in patients with sigmoid volvulus and related statistical analyses.

Parameter	Nonoperative treatment	Emergency surgery	Statistical analysis
Total	603	492	-
Evaluated	533	492	-
Previous volvulus history	185/533 (34.7%)	129/492 (26.2%)	x^2^=8.678, p=0.003^a^
Pregnancy	5/92 (5.4%)	5/90 (5.6%)	x^2^=0.001, p=0.971^a^
Comorbidity	169/533 (31.7%)	143/492 (29.1%)	x^2^=0.844, p=0.358^a^
Symptom period (hours)	23.9±13.8	47.5±30.4	t=16.205, p<0.001^b^
Abdominal pain/tenderness	527/533 (98.9%)	487/492 (99.0%)	x^2^=0.0.29, p=0.865^a^
Distention	518/533 (97.2%)	484/492 (98.4%)	x^2^=1.647, p=0.199^a^
Obstipation/constipation	495/533 (92.9%)	466/492 (94.7%)	x^2^=1.488, p=0.223^a^
Vomiting	334/533 (62.7%)	370/492 (75.2%)	x^2^=18.701, p<0.001^a^
Hypokinetic/akinetic bowel sound	199/533 (37.3%)	236/492 (48.0%)	x^2^=11.838, p<0.001^a^
Hyperkinetic bowel sound	188/533 (35.3%)	99/492 (20.1%)	x^2^=29.128, p<0.001^a^
Empty rectum	362/533 (67.9%)	296/492 (60.2%)	x^2^=6.694, p=0.010^a^
Gangrenous stool	0/533 (0.0%)	96/492 (19.5%)	x^2^=114.747, p<0.001^a^
Rebound tenderness/muscular rigidity	0/533 (0.0%)	81/492 (16.5%)	x^2^=95.279, p<0.001^a^
Shock	7/533 (1.3%)	109/492 (22.2%)	x^2^=110.723, p<0.001^a^

a Chi-square test,

b Student t test

As demonstrated in Table-I, the rate of previous volvulus history was statistically higher in nonoperative detorsion group when compared with that of the emergency surgery group (34.7% vs. 26.2%, respectively, P<0.005). However, in nonoperative detorsion and emergency surgery groups, pregnancy (5.4% vs. 5.6%, respectively, p>0.05) and comorbidity (31.7% vs 29.1%, respectively, p>0.05) rates were statistically similar. Mean symptom period was statistically shorter in nonoperative detorsion group when compared with that of the emergency surgery group (23.9 hours vs. 47.5 hours, respectively, p<0.001). The rates of abdominal pain/tenderness (98.9% vs. 99.0%, respectively, p>0.05), distention (97.2% vs. 98.4%, respectively, p>0.05) and obstipation/constipation (92.9% vs. 94.7%, respectively, p>0.05) were statistically similar in nonoperative detorsion and emergency surgery groups. However, when compared with that of the emergency surgery group, the rates of vomiting (62.7% vs. 75.2%, respectively, p<0.001) and hypokinetic/akinetic bowel sound were lower (37.3% vs. 48.0%, respectively, p<0.001), while hyperkinetic bowel sound (35.3% vs. 20.1%, respectively, p<0.001) and empty rectum were higher (67.9% vs. 60.2%, respectively, p<0.05) in nonoperative detorsion group. On the other hand, all patients with gangrenous stool (19.5% vs. 0.0%, respectively, p<0.001) and rebound tenderness/muscular rigidity (16.5% vs. 0.0%, respectively, p<0.001) required emergency surgery. Finally, shock rate was statistically lower in nonoperative detorsion group when compared with that of emergency surgery group (1.3% vs. 22.2%, respectively, p<0.001).

## DISCUSSION

Although first line treatment is nonoperative detorsion in SV, requirement for emergency surgery may run into 69.3% in relatively large series.^5^,^6^ However, emergency surgery worsens the prognosis with 1%-30% of mortality and 5%-60% of morbidity rates.^3^,^7^,^8^ For this reason, some preoperational parameters including clinical features may set light to treatment planning.

In the present series, we demonstrated relatively high rates of previous SV history, which are indicators of high SV recurrence. SV is a repetitive clinical entity unless one performs definitive surgery.^9^-^11^ In a 54-case series, Memis and Aydin^12^ reported previous SV history rate as 29.6%, which is similar to our results. Interestingly, recurrence rate was higher in nonoperative treatment group (34.7%) when compared with that of emergency surgery group (26.2%) in our series. In our experience, multiple SV attacks causes fibrosis and narrowing in sigmoid mesentery, which induces following attacks. However, this anatomic stricture also eases spontaneous or endoscopic detorsion. For this reason, patients with previous SV attacks, particularly with multiple torsions, frequently response to endoscopic detorsion.

SV is the most common type of intestinal obstructions in pregnant women, which constitutes 44% of all intestinal obstructions in pregnancy.^13^ SV complicating pregnancy is not an absolute indication of emergency surgery as was demonstrated in our series. Recent reports encourage practitioners to apply endoscopic detorsion instead of emergency surgery in pregnant women with SV.^14^,^15^ High rate of comorbidity is an unavoidable situation in SV due to the disease generally affects elderly people.^1^,^3^ According to our results, comorbidity is not an effective factor in selection of treatment options. Gul et al.^16^ reported a relatively high comorbidity rate (56.8%) in their surgically treated 44-case series. However, Dahiya et al.^17^ reported a negative correlation between high Charlson comorbidity index and requirement for emergency surgery in 90,902-patient multicenter group with colonic volvulus. Similarly, Deresse et al.^18^ reported 5.3% of comorbidity rate and Emna et al.^19^ presented 6.3% of high (≥4) American Society of Anesthesiologists score in their emergency surgically treated 170 and 64 SV cases.

Our results demonstrated a positive correlation between late admission and requirement for emergency surgery. Similarly, in emergency surgically treated 170- and 131-patients series, Deresse et al.^18^ (all emergency surgery) and Mulugeta and Awlachew^20^ (mostly emergency surgery) demonstrated 68.2% and 54.2% late admission (>24hours) rates, respectively. In other studies, Pattanaik^9^ (mostly emergency surgery) and Emna et al.^19^ (all emergency surgery) reported mean preoperative period as 72 hours and 4.2 days, respectively, in 366- and 64-patient groups. In clinical practice, prolonged preoperational period makes endoscopic detorsion difficult due to intraluminal gas expansion in time. However, late admission is not the sole effective factor necessitating emergency surgery and some other agents such as over-rotation or vascular anatomical variations may trigger bowel gangrene even if the symptom period is not long enough.

Abdominal pain/tenderness is one of the main clinical features of SV. Although it was present in almost all of our patients, we could not find it as an indicator of emergency surgery. Fo et al.^21^ and Agrawal^22^ demonstrated pain/tenderness in all of emergency surgically treated 9 and 27 cases, respectively. Similarly, in different series, Pattanaik^9^, Memis and Aydin^12^ and Mulugeta and Awlachew^20^ found this rate as 84.8%, 92.6% and 100%, respectively, in 366-, 54- and 131-case series, respectively, in which emergency surgery was the most preferred treatment option. On the other hand, Iida et al.^23^ identified the presence of abdominal tenderness as a factor predicting unsuccessful endoscopic detorsion and necessity for emergency surgery in 21-patient study.

Abdominal distention is another important clinical symptom and sign of SV, which did not vary between treatment groups in our study. In literature, the rate of this clinical feature was found as 100% by Pattanaik^9^ (366 patients, mostly emergency surgery), by Mulugeta and Awlachew^20^ (131 cases, mostly emergency surgery) and by Agrawal^22^ (27 cases, all emergency surgery). Similarly, in some other patient populations, this rate was presented as 96.3% by Memis and Aydin^12^ (54 cases, mostly emergency surgery), 94.1% by Deresse et al^18^ (170 patients, all emergency surgery). It is clear that abdominal distention generally increases in time. Although excessive distention is not an absolute indication of emergency surgery, it may be an impediment to endoscopic detorsion.

Obstipation/constipation is the third most common clinical feature of SV, which was demonstrated in similar rates between nonoperative detorsion and emergency surgery groups in the present study. Pattanaik^9^ (366 patients, mostly emergency surgery), Mulugeta and Awlachew^20^ (131 cases, mostly emergency surgery) and Agrawal^22^ (27 cases, all emergency surgery) presented inability to pass feces and flatus in all of their patients with SV. On the other hand, this rate was reported to be 59.3% by Memis and Aydin^12^ (54 cases, mostly emergency surgery), 56.8% by Gul et al.^16^ (44 cases, all emergency surgery) and 84.7% by Deresse et al.^18^ (170 patients, all emergency surgery). Although residual feces and gas in the distal part of the obstruction line may cause limited number and amount of defecation or degassification, obstipation is one of the main clinical features of SV and some practitioners describe it as constipation. However, in our experience, continuous diarrhea, particularly long-standing form, may be a premorbid symptom triggering volvulus, which is generally confused with primary features of SV.

Although vomiting appeared as a factor raising the requirement for emergency surgery in our study, it is not an absolute indication of emergency surgery. However, prolonged preoperational period increases the rate of vomiting incidence due to accumulation of the gastrointestinal content. In emergency surgically treated 170- and 9-case series, Deresse et al.^18^ and Fo et al.^21^ declared 61.2% and 66.7% of vomiting rates, respectively. Conversely, Pattanaik^9^, Mulugeta and Awlachew^20^ and Memis and Aydin^12^ reported vomiting rates as 18.7%, 19.8% and 22.2% in their 366-, 131- and 54-case series, respectively, in which emergency surgery was the main treatment choice.

Hyperkinetic bowel sound is a common finding of uncomplicated SV. However, bowels get tire in time and hypokinetic/akinetic bowel sounds substitute for hyperkinesia, which may be a relative precursor of emergency surgery, as was seen in our study. In a 366-patient SV series, most of whom treated with emergency surgery, Pattanaik^9^ found hypertympanic bowel sound in 88.4% of the cases, while reduced or absent bowel sounds were reported to be 45.3%.

Findings of digital rectal examination have great importance in SV. Although a little amount of feces may be determined in some early cases, empty rectum is a common finding of medium-term SV. However, gangrenous stool is an absolute indication of emergency surgery, particularly in delated cases, as was presented in our data. Agrawal^22^ presented empty rectum in all of 27 patients with emergency surgery. Pattanaik,^9^ and Mulugeta and Awlachew^20^ reported 80.3% and 69.4% of empty rectum rates among 366- and 131-case SV series, respectively, most of the patients required emergency surgery. In the later series, gangrenous material was found in digital rectal examination in 9.9% of the cases. It is well known that bowel gangrene is an undesirable complication of SV, which is reported to be 56.3% by Deresse et al.^18^ and 18.5% by Moro-Valdezate et al.^24^ in relatively large series.

Rebound tenderness/muscular rigidity is another fearful finding of SV, which generally indicates acute abdomen and requires emergency surgery, as was seen in our series. Surek et al.^25^ reported 69.9% of abdominal tenderness rate in their surgically treated 52 patients. In another 366-case series, in which most of the patients required emergency surgery, Pattanaik^9^ reported rebound tenderness rate as 7.5%.

Shock is frequently seen in delated cases, which worsens the prognosis. In our series, we frequently determined shock in cases with late admission or with bowel gangrene, which patients generally required emergency surgery. Toxic, septic, or hypovolemic shock rates are reported to be 15.1% by Pattanaik^9^, 11.7% by Deresse et al.^18^ and 9.8% by Moro-Valdezate et al.^24^, most of the cases treated with emergency surgery in above-mentioned series.

### Limitations:

Despite the evaluation of a relatively large SV series, the usage of combined (retrospective and prospective) consideration and non-matched statistical analysis were the major limitations of this study. It should not be forgotten that, single-center prospective evaluation requires hundreds of SV cases during several decades.

## CONCLUSIONS

In conclusion, gangrenous stool in rectal examination and rebound tenderness/muscular rigidity are absolute indications of emergency surgery, while first attack, prolonged preoperative period, vomiting, hypokinetic/akinetic bowel sound and shock generally requires emergency surgery. On the other hand, we could not find any positive correlation between pregnancy, comorbidity, abdominal pain/tenderness, distention and obstipation/constipation and requirement for emergency surgery.
